# The presence, severity, and onset of preeclampsia is associated with maternal interleukin-23 level: A case-control study

**DOI:** 10.18502/ijrm.v21i4.13269

**Published:** 2023-05-08

**Authors:** Forough Forghani, Nasrin Ranjbar, Danial Jahantigh

**Affiliations:** ^1^Department of Obstetrics and Gynecology, Zabol University of Medical Sciences, Zabol, Iran.; ^2^Department of Obstetrics and Gynecology, Zahedan University of Medical Sciences, Zahedan, Iran.; ^3^Department of Clinical Biochemistry, Zahedan University of Medical Sciences, Zahedan, Iran.; ^4^Department of Biology, Faculty of Science, University of Sistan and Baluchestan, Zahedan, Iran.

**Keywords:** Preeclampsia, Interleukin-23, Pregnancy, Inflammation.

## Abstract

**Background:**

Scientific evidence support that imbalance between inflammatory and anti-inflammatory cytokines play a critical role in preeclampsia (PE).

**Objective:**

To investigate the relationship between the maternal serum level of interleukin (IL)-23, a pro-inflammatory cytokine, PE and its severity risk was investigated.

**Materials and Methods:**

The case-control study included a total of 145 women counting 75 PE cases, 35 healthy pregnant and 35 healthy non-pregnant controls from Zahedan, southeast of Iran. The maternal levels of IL-23 in circulation were determined via enzyme-linked immunosorbent assay.

**Results:**

The maternal serum levels of IL-23 were increased in PE and its 2 subgroups severe PE and mild PE, so that these increases were significant in PE and severe PE, but not in mild PE compared with the controls (p < 0.001 and p < 0.001, p = 0.08, respectively). Besides, the maternal IL-23 serum level was statically significant in the early onset PE, but not in the late onset-PE group compared to healthy pregnant controls (p < 0.001, p = 0.46 respectively).

**Conclusion:**

The results of our study showed a positive association between IL-23 level and PE, especially in severe type and early onset PE, which suggests that IL-23 may be involved in the pathogenesis of this systemic syndrome.

## 1. Introduction 

In pregnancy, preeclampsia (PE) is considered to be a specific disorder that can threaten the life of both the mother and the fetus in 3-5% of pregnant women (1). The symptoms become apparent between the second and third trimester of gestation with a placental disorder caused by unusual vascular reply to placentation, which can lead to endothelial cell dysfunction and systemic vasospasm (2). Edema, renal failure, and hemolysis, elevated liver enzymes and low platelets syndrome are some of the additional symptoms that similarly exacerbate the clinical condition. However, the clinical signs and diagnostic standards for PE include high blood pressure, proteinuria, abrupt overweight, edema, liver enzymes elevation, renal impairment, and blurred vision (1, 2). PE causes fetal growth restriction (FGR), weight loss, and eventually perinatal death (3). The PE might have mild or severe symptoms with early or late onset of clinical manifestations. Severe cases of PE are diagnosed with symptoms such as unbalanced liver function, thrombocytopenia, and hemolysis, elevated liver enzymes and low platelets syndrome. Although the etiology of PE is not exactly identified, it is generally established that both maternal and placental factors are related to its etiology and pathogenesis (3, 4).

Inflammatory cytokines are recognized as powerful stimulators of the vascular endothelium and inflammatory mediators of endothelial cell dysfunction (4). Briefly, placentae in stressful circumstances release a surplus of cytokines that play a role in maternal endothelial cell activation and inflammatory replies that are indicators of PE (5). Different cytokines were detected in PE in comparison to healthy pregnant women; therefore, the high level of cytokines include granulocyte-macrophage colony-stimulating factor, colony-stimulating factor-1, interleukin (IL) 3, and IL-10 that have positive effects on successful pregnancy, while IL-1β, IL-2, IL-6, IL-8, IL-17, IL-27 and tumor necrosis factor-alpha (TNF-α), and interferon-gamma (IFN-γ) contributed well to PE (4-6).

IL-23, a pro-inflammatory cytokine, is structurally similar to IL-6 and IL-12 which belong to the IL-12 family. IL-23 is considered an IFN-γ production inducer and is a crucial cytokine in the regulation of the development of Th17 cells, which prompts high levels of the pro-inflammatory cytokine IL-17 (7), as well as convinces the production of other inflammatory cytokines, including TNF-α, IL-1, IL-6, and IL-8 (8). Furthermore, IL-17 which is secreted via IL-23/ T-helper type 17 (Th17) axis, is suggested as a hypertension inducer during pregnancy (9). The important role of IL-23 has been detected in many disorders including psoriasis, multiple sclerosis, systemic lupus erythematosus, rheumatoid arthritis, inflammatory bowel disease, hepatitis C virus infection, and Coronavirus disease 2019 (10-18).

A limited recent study reported increased levels of Th17/Treg cytokines profile, including IL-23, in PE (19), but the association between IL-23 levels and the presence, severity, and onset of PE remains controversial. This study focuses on comparing the IL-23 serum level in PE with 2 different control groups and, investigates the susceptibility to PE complicated by FGR in an Iranian population for the first time in the literature.

## 2. Material and Methods

25 participants were considered as the minimum number of participants that was computed through a comparison of the mean difference between the 2 groups regarding the following assumptions: 80% power, 95% CI, 1:1 PE: controls ratio. The present case-control study was done on 145 subjects referring to Imam Ali hospital, Zabol, Iran from July 2017 to November 2018. The PE and controls were 112 and 70 subjects, respectively. 37 cases were excluded from the study because of the following reasons that were described previously (20): twin or multiple pregnancies, tuberculosis, leprosy, tularemia, sepsis, lyme disease, liver diphtheria, renal disease, diabetes, hydrops fetalis, hydatidiform mole.

The control group had a normal pregnancy without any fetal disorders, pathological problems, and no history of multiple births, as well. None of the control subjects shown PE after giving birth.

The age-matched healthy controls were excluded due to common autoimmune diseases. No history of hypertension was detected in the PE and control groups.

The women with systolic blood pressure > 140 mmHg and/or diastolic blood pressure > 90 mmHg, and also, proteinuria > 300 mg in a 24 hr period or > 1+, after 20 W of pregnancy sorted in PE group. Both cases and controls contain different subgroups, so that, on one hand, PE is divided into 46 severe and 29 mild PE groups. The controls comprise 35 healthy pregnant and 35 healthy non-pregnant women (Table I). The diagnostic criteria of severe PE, mild PE, early and late-onset were described in our previous works (6, 20). The PE subjects which have high blood pressure ≥ 160 mmHg systolic or ≥ 110 mmHg diastolic and also, protein excretion ≥ 5 g/24 hr were characterized as severe PE. Moreover, the case group was assigned into 2 other subgroups early-onset PE ( ≤ 34 wk of gestation, n = 34) and late-onset PE ( > 34 wk of gestation, n = 41) (Tables II, III). Blood samples were stored at -80 C until further usage. IL-23 serum levels were measured in PE and control women using an ELISA kit (Bio Legend, San Diego, CA, USA), pursuant to the manufacturer's instruction. The sensitivity of the IL-23 test was 4.27 pg/mL in our laboratory.

### Ethical considerations 

The Ethics Committees of Zabol University of Medical Sciences, Zabol, Iran approved this study (Code: Zbmu.1.REC.1396.25). All subjects signed an informed consent form before participation, as well.

### Statistical analysis 

All the records obtained were mean ± SD. The Kolmogrov-Smirnov test was recruited to test the normal distribution of data. The ANOVA and Student *t* tests statistical analysis were performed for normally distributed data, and/or Kruskal-Wallis and Mann-Whitney U tests were utilized for non-normally distributed data via GraphPad Prism (version 7.01) software package (Graph Pad, CA, USA). The p < 0.05 was contemplated statistically significant.

## 3. Results

The clinical properties of cases and controls are summarized in table I. The average weekly pregnancy in the patient group was 33.71 ± 5.36, which was not significantly different from the control group. In terms of age and body mass index, no significant difference was observed between healthy and pregnant women. However, proteinuria, systolic blood pressure, and diastolic blood pressure were significantly different in the healthy group and the patient group (p < 0.001). In addition, the birth weight of the newborns in the healthy group was higher than that of the PE group, the result of which is shown in table I.

The maternal serum levels of IL-23 significantly increased in PE and healthy pregnant groups which compared to non-pregnant controls (p < 0.001 and p < 0.001, respectively). Moreover, a significantly elevated amount of IL-23 was observed in PE women compared with healthy pregnant women, with the median of 24.461 ± 8.027 pg/ml compared with median of 17.622 ± 6.310 pg/ml, respectively (p < 0.001). Also, when the PE women were divided into different severe and mild PE subgroups, the IL-23 serum levels remained significantly higher in severe PE than in healthy pregnant women (p < 0.001). No significant association was observed between mild PE and healthy pregnant women (p = 0.08).

A highly significant difference was observed between severe PE women and mild PE groups in IL-23 serum level: median of 26.991 ± 7.989 vs. the median of 20.448 ± 6.363 pg/ml, respectively (p < 0.001). According to the onset of PE, a significant difference was observed in IL-23 levels between early-onset PE and late-onset PE (p < 0.001). Moreover, when the PE women were sorted into 2 PE women with or without FGR, the FGR PE group showed a higher level of IL-23, but this increase was not significant, (Table II, p = 0.29).

Based on the PE severity, we classified early and late-onset PE into severe and mild types. As shown in table III, the IL-23 levels were significantly different in early-onset PE, but not late-onset PE compared to gestation-matched healthy pregnant controls (p < 0.001 and p = 0.46, respectively). A highly significant elevation of the IL-23 serum level was found in early-onset PE in both severe and mild type and gestation-matched healthy pregnant controls (p < 0.001). Interestingly, no statistically significant association was observed in mild and severe PE in late-onset in comparison to gestation-matched healthy pregnant (Table III, p = 0.92 and p = 0.22, respectively).

**Table 1:** Demographic characteristics of the study population


**Variables **	**Healthy non-pregnant women (n = 35)**	**Healthy pregnant women (n = 35)**	**PE (n = 75)**	P-value ${}^{*}$	P-value ${}^{**}$
**Age (yr)**	26.44 ± 7.05	27.31 ± 4.82	27.93 ± 7.11	0.30	0.64
**BMI (kg/m^2^)**	24.76 ± 5.52	26.03 ± 6.14	29.19 ± 6.23	< 0.001	0.001
**Gestation at sample (wk)**	NA	33.35 ± 3.89	33.71 ± 5.36	- 0.72
**Systolic pressure (mmHg)**	109.94 ± 11.34	112.62 ± 10.17	152.78 ± 13.76	< 0.001	< 0.001
**Diastolic pressure (mmHg)**	66.52 ± 9.45	65.21 ± 7.54	96.63 ± 9.84	< 0.001	< 0.001
**Proteinuria**	0	0	+ or more	< 0.001	< 0.001
**Birthweight (g)**	NA	3244.61 ± 412.66	2405.13 ± 1096.51	- < 0.001
Data was presented as Mean ± SD. The results of ANOVA statistical analysis revealed that demographic data had normally distributed, so, student *t* tests analysis were performed for all of them. PE: Preeclampsia, BMI: Body mass index, NA: Not applicable, ${}^{*}$ Healthy non-pregnant women vs PE, ${}^{**}$ Healthy pregnant women vs PE					

**Table 2:** The serum levels of IL-23 (pg/ml) in PE and normal pregnant women


**Group**	**Number**	**IL-23 levels**	**P-value**
**Healthy pregnant women**	35	17.622 ± 6.310	< 0.001
**PE**	75	24.461 ± 8.027	< 0.001
**Healthy non-pregnant women**	35	12.702 ± 4.155	Ref.
**PE**	75	24.461 ± 8.027	< 0.001
**Severe PE**	46	26.991 ± 7.989	< 0.001
**Mild PE**	29	20.448 ± 6.363	0.08
**Healthy pregnant women**	35	17.622 ± 6.310	Ref.
**Severe PE **	46	26.991 ± 7.989	< 0.001
**Mild PE **	29	20.448 ± 6.363	Ref.
**Early-onset PE**	34	29.532 ± 7.192	< 0.001
**Late-onset PE **	41	20.256 ± 6.044	Ref.
**PE with FGR **	17	26.841 ± 4.684	0.29
**PE without FGR **	58	24.669 ± 8.168	Ref.
Data was presented as Mean ± SD. The results of Kruskal-Wallis statistical analysis shown that IL-23 serum level was non-normally distributed, so, Mann-Whitney U tests analysis were performed for all of them. IL: Interleukin, PE: Preeclampsia, FGR: Fetal growth restriction			

**Table 3:** The serum levels of IL-23 (pg/ml) in early-onset, late-onset PE and gestation-matched normal pregnant women


**Group**	**Number**	**IL-23 levels **	**P-value**
**Early-onset PE**	34	29.532 ± 7.192	< 0.001
**Severe PE **	20	33.450 ± 6.893	< 0.001
**Mild PE**	14	23.936 ± 2.126	< 0.001
**Healthy pregnant women (≤ 32 wk of gestation)**	16	16.138 ± 5.260	
**Late-onset PE**	41	20.256 ± 6.044	0.46
**Severe PE**	23	19.074 ± 6.199	0.92
**Mild PE**	18	21.767 ± 5.649	0.22
**Healthy pregnant women (> 32 wk of gestation)**	19	18.8716 ± 8.243	
Data was presented as Mean ± SD. The Kruskal-Wallis and Mann-Whitney U tests were utilized. IL: Interleukin, PE: Preeclampsia			

## 4. Discussion

The results of the current study showed a high level of IL-23 in severe PE women, which is in assent with previous reports that showed higher levels of inflammatory cytokines in women with severe PE. Moreover, the highest level of IL-23 was seen in early severe PE as compared to gestation-matched healthy group, which is consistent with some studies, but not with others in the relationship between inflammatory cytokines and onset of PE (4-6). In addition, we found a significant association in IL-23 levels between early and late-onset PE with elevated amounts of serum level in the severe and mild types of early-onset PE.

PE can be assumed as a response to the increased production of inflammatory cytokines; one of the possible causes of PE increase is the imbalance of pro-inflammatory and anti-inflammatory cytokines (1, 2). Different expression of inflammatory and anti-inflammatory cytokines profile was reported in PE in healthy pregnant controls (5, 6). Moreover, it has been shown that severe PE has higher levels of inflammatory cytokines including IL-6, IL-12, and IL-27 along with the reduction of IL-5, and IL-10 compared to healthy pregnant controls, proposed that inflammatory cytokines may be considered critical factors in the incidence and severity of PE (4-6).

Several studies consider IL-23 as an inflammatory cytokine that mediated various immune disorders (10-18); thus we hypothesized that the serum IL-23 level might be associated with PE or its severity. In this study, we detected a significant association between IL-23 levels of PE and healthy pregnant women compared with non-pregnant controls (Table II). Furthermore, it can be proposed that the serum IL-23 levels were positively correlated with the severity and onset of PE. Accordingly, elevated IL-23 levels were observed in severe type as well as early onset PE (Table III).

IL-23, as a member of the family IL-12, released by macrophages and dendritic cells activated and can induce and produce IFN-γ, but IL-23 has a distinctive capacity at IL-17 production induction in comparison with IL-12, which happened in inflammatory diseases (7). In addition, IL-23 is essential in the development of Th17, as well as has a pivotal role in the survival and expansion of pathological Th17 cells (7, 21). Through IFN-γ production, IL-23 causes increased activity of trophoblast CD4 ${}^{+}$ cells, these cells produce IL-17, IL-6, TNF-α, and other inflammatory cytokines (21, 22). Previous studies have recognized IFN-γ as a major reason for PE, which has a critical role in the expansion of inflammatory cytokines. Accumulating data propose that IFN-γ-producing cells have a pivotal impact on immune system disorders such as PE (8, 23). In addition, it has been confirmed that IL-23-dependent T cells are highly pathogenic and has an essential role in inflammatory responses (21, 23).

The participation of TH17 cells in pregnancy-related disorders including PE has been revealed lately. In these conditions, TH17 cells have been adjusted so that the prevalence of TH17 cells in PE women can be seen (8, 19, 24, 25). As a result, the positive induction of TH17 cells by increasing the amount of IL-23 leads to the production of IL-17 cytokines, further releasing other cytokines such as IL-6 that cause systemic inflammation in PE.

Because of the unique features of IL-23 as an important autoimmune and inflammatory pathology agent, IL-23 can be considered as a new therapeutic goal. Therefore, the clinical progression of some of the IL-23 receptor antagonists, by encouraging it, can result in numerous autoimmune inflammatory diseases (10-16). Our finding suggested that IL-23 might have an important role in the development of PE, and if these findings are confirmed with more extensive studies, IL-23 can be considered as a new therapeutic goal for PE.

## 5. Conclusion

IL-23 serum levels in PE, and also in the severe, mild, early-onset and late-onset subgroups have been analyzed. Our results showed a significant association between serum levels of IL-23 and total PE. Furthermore, other severe and early-onset PE subgroups revealed a significantly increased IL-23 cytokine level. Further studies are needed to confirm our findings; however, the findings of our study indicate that the IL-23 level can help PE and potentially consider it as a new marker for its diagnosis.

##  Conflict of Interest

The authors declare that they have no competing interest.
